# Remnant cholesterol, lipid ratios, and the severity of coronary artery lesions: a retrospective cohort study in patients with coronary heart disease

**DOI:** 10.3389/fcvm.2025.1516326

**Published:** 2025-03-10

**Authors:** Yu Li, Yumei Zhai, Songli Hu, Jing Liu, Wenchen Zhang, Jianwei Yue, Zichao Wang

**Affiliations:** Institute of Hypertension Research, The Second Affiliated Hospital of Baotou Medical College, Inner Mongolia University of Science and Technology, Baotou, China

**Keywords:** remnant cholesterol, lipid ratios, coronary heart disease, coronary artery lesions, Gensini score

## Abstract

**Background:**

Emerging genetic and observational evidence indicates that remnant cholesterol (RC) is a significant residual risk factor for cardiovascular diseases. However, there is a relative paucity of evidence exploring the correlation among RC, lipid ratios, and atherosclerotic lesion severity. This study aimed to investigate the predictive value of RC and lipid ratios alone or in combination for the severity of coronary artery stenosis in patients with coronary heart disease (CHD).

**Methods:**

The Gensini score was used to assess the severity of coronary atherosclerotic lesions. CHD patients were categorized into mild stenosis and moderate-to-severe stenosis groups. Logistic regression was used to evaluate the risk of a high Gensini score associated with RC and lipid ratios. Our study also examined the relationship between inconsistencies in RC and non-high-density lipoprotein cholesterol (non-HDL-C) levels and the severity of coronary artery stenosis. Receiver operating characteristic (ROC) curves were used to assess the predictive power of RC and lipid ratios alone or in combination for moderate to severe coronary artery lesions.

**Results:**

Multivariate regression models suggested that RC was a strong predictor of moderate to severe coronary artery stenosis [odds ratio (OR): 5.44, *P* < 0.001]. When grouped by curve-fitting inflection points, the group with inconsistent high RC/low non-HDL-C, rather than the low RC/high non-HDL-C group, was associated with an increased risk of moderate to severe coronary stenosis compared with the consistent low RC group (OR: 2.72, *P* < 0.001). ROC curves showed that RC predicted an area under the curve (AUC) of 0.715 for coronary stenosis severity, improving the predictive efficacy of the combined predictors comprising lipid ratios (AUC: 0.723 vs. 0.703, *P* < 0.05).

**Conclusions:**

RC and various lipid ratios [triglyceride/HDL-C, total cholesterol/HDL-C, low-density lipoprotein cholesterol/HDL-C, and apoloprotein (apo)B/apoA] correlated with the degree of coronary artery stenosis in patients with CHD, suggesting that RC has potential value as a biomarker reflecting the degree of coronary artery stenosis independent of the traditional risk factors and the levels of non-HDL-C. This could enhance the predictive efficacy based on the lipid ratio model and had better predictive value for moderate to severe coronary artery lesions.

## 1 Introduction

Coronary heart disease (CHD) is a cardiovascular disease (CVD) with high morbidity worldwide, featuring rapid changes in conditions and high mortality, as well as high disability and poor prognosis. It is a serious threat to the life and health of patients. CHD is based on atherosclerosis (AS), in which vulnerable plaques rupture and form thrombi, causing coronary artery stenosis and occlusion, ultimately leading to myocardial infarction (MI) (1). Therefore, timely recognition of the early signs of CHD and accurate prediction of the severity of coronary lesions are essential for its prevention, treatment, and prognosis.

Dyslipidemia is a well-established pathophysiological link between atherosclerosis and vascular stenosis (2). Studies have shown that dyslipidemia, particularly elevated low-density lipoprotein cholesterol (LDL-C), is an independent risk factor for CHD (3–5). Currently, LDL-C is the cornerstone of traditional and most well-established lipid-lowering therapies, with most clinical guidelines identifying LDL-C level as the primary target for the CHD prevention and treatment (6). However, its clinical application presents certain challenges. First, direct methods for detecting LDL-C are limited by complex procedures and high costs, and fluctuations in triglyceride (TG) levels can significantly affect the accuracy of LDL-C estimation using the Friedewald formula (7). However, existing studies have indicated that even with intensified lipid-lowering therapy, including both pharmacological treatment and lifestyle interventions to achieve target LDL-C levels and control other traditional risk factors, patients with CHD still face a higher risk of major adverse cardiovascular events (MACEs) (8, 9). This persistent risk in the context of controlled LDL-C is defined as “residual cardiovascular risk,” emphasizing the need to focus on emerging biomarkers, such as remnant cholesterol (RC), lipoprotein(a) [Lp(a)], and high-sensitivity C-reactive protein, in addition to controlling traditional indicators like LDL-C and TG. These biomarkers play a role in the occurrence, progression, and prognosis of CVDs.

Studies have shown that high levels of RC are closely related to CVDs such as ischemic heart disease (IHD) (10, 11), peripheral arterial disease (PAD) (12, 13), and hypertension (14, 15). RC includes cholesterol from all triglyceride-rich lipoproteins (TRLs), including very low-density lipoprotein cholesterol (VLDL-C), intermediate-density lipoprotein cholesterol (IDL-C) when fasting, and celiac microparticles (CM) when not fasting (16). Current evidence indicates that RC, an emerging lipid marker, is associated with CHD prognosis, with elevated RC levels significantly increasing the risk of cardiovascular events and all-cause mortality in patients with CHD (17–19). However, the relationship between RC and severity of coronary artery stenosis in patients with CHD remains unclear. Lipid ratios can directly reflect the balance between atherogenic and protective factors, theoretically providing a more accurate assessment of the extent of coronary artery lesions than the individual components. Currently, research on the predictive value of RC and lipid ratios for coronary artery stenosis severity is limited.

Therefore, our study used the Gensini score to assess the severity of coronary lesions, aiming to explore the individual or combined assessment roles of RC and lipid ratios in determining the severity of coronary lesions in patients with CHD. Additionally, we explored the correlation between the inconsistency of RC and non-high-density lipoprotein cholesterol (non-HDL-C) levels, and the severity of coronary artery lesions in patients with CHD to complement the independent predictive value of RC for coronary lesions.

## 2 Materials and methods

### 2.1 Study participants

**Figure 1:**
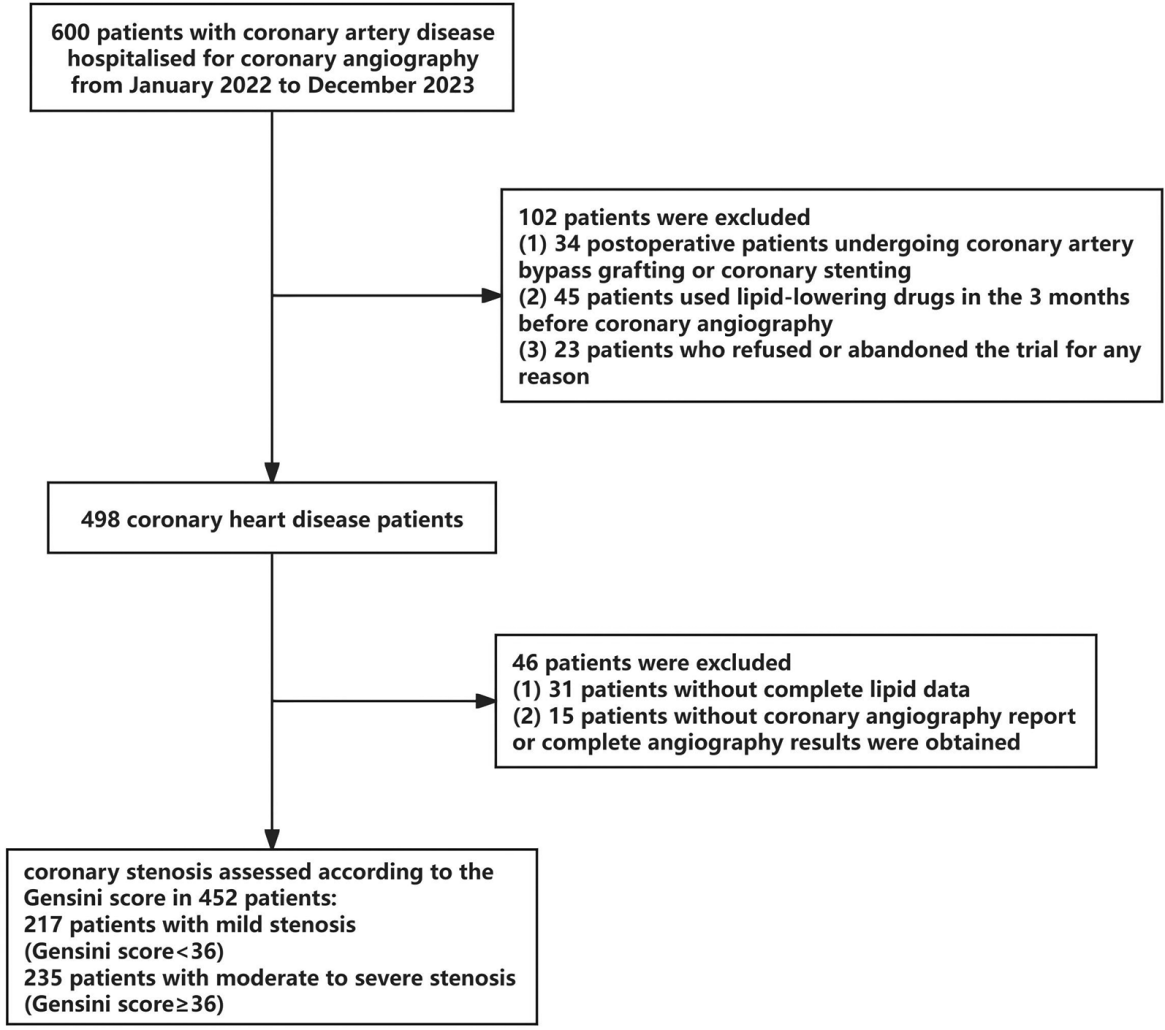
Flowchart of patients inclusion processing.

The clinical data of 600 patients with CHD who were admitted to the Department of Cardiology of the Second Affiliated Hospital of Baotou Medical College for coronary angiography owing to chest tightness and chest pain between January 2022 and December 2023 were retrospectively analyzed, and 452 patients were finally determined to be study participants, following the exclusion of 102 patients who did not meet the inclusion criteria and 46 patients with incomplete baseline data (Figure 1). Inclusion criteria were: (1) age ≥18 years; (2) no previous use of lipid-lowering drugs or irregular use and no use in the past 3 months; (3) underwent coronary angiography or coronary intervention to clarify the stenosis of blood vessels; (4) newly diagnosed with CHD according to the relevant diagnostic criteria of the guideline for the diagnosis and treatment of CHD (20); (5) voluntarily participated in this study. Exclusion criteria were: (1) having incomplete clinical data; (2) having severe heart valve disease, intractable heart failure, cardiomyopathy, or other heart diseases; and (3) having malignant tumors, severe systemic infections, severe liver and renal insufficiency, and acute cerebrovascular accidents.

Our study complied with the ethical requirements of the Declaration of Helsinki and its subsequent revisions and had been reviewed and approved by the Ethics Committee of the Second Affiliated Hospital of Baotou Medical College (Ethics Review Approval Number: 2024-ZX-047), and this study had obtained written consent from all study participants.

### 2.2 Data collection

Basic information was collected from all enrolled patients, including sex, age, height, weight, smoking status (defined as an average of at least one cigarette per day for a minimum of one year, currently smoking, or having quit for less than one year at the time of admission), alcohol intake (defined as averaging at least one alcoholic drink per day over the past year with a daily intake exceeding 50 ml), medical history (including hypertension, diabetes mellitus, and hyperlipidemia), and family history of CHD. Hypertension is defined as systolic blood pressure ≥140 mmHg and/or diastolic blood pressure ≥90 mmHg on three separate occasions without taking antihypertensive medication (21). According to the latest diabetes treatment guideline of the American Diabetes Association, the diagnostic criteria for diabetes are fasting blood glucose ≥7.0 mmol/L, glycated hemoglobin ≥6.5%, or 2-h blood glucose during oral glucose tolerance test or random blood glucose ≥11.1 mmol/L, any of which can diagnose diabetes (22).

### 2.3 Lipid measurements

All patients underwent fasting venous blood collection within 24 h of admission while in a resting state. Biochemical parameters, including lipid profile, fasting blood glucose (FBG), N-terminal precursor of brain natriuretic peptide (NT-proBNP), and troponin I (TnI), were measured in the central laboratory of our hospital on the same day as blood collection. TG and total cholesterol (TC) levels were determined using immunoturbidimetry with specific antibodies. HDL-C was measured directly by chemical modification without the need to isolate other lipoproteins. LDL-C was quantified using a selective solubilization method that selectively dissolves other lipoproteins (such as HDL and VLDL), and plasma cholesterol content was measured enzymatically. Apolipoprotein A (ApoA) and apolipoprotein B (ApoB) levels were determined using immunoturbidimetry. Fasting blood glucose levels were measured using the glucose assay. Each blood sample measurement was repeated and the average values were calculated. This study utilized a Siemens ADVIA series automated biochemical analyzer (ADVIA 1800) for efficient and accurate detection of lipid and glucose levels. All patients underwent echocardiography to record left ventricular ejection fraction (LVEF).

Based on the results of blood lipid tests, used formulas to calculate the results of RC, non-HDL-C, where RC = TC-HDL-C-LDL-C, mmol/L (17), non-HDL-C = TC-HDL-C, mmol/L (23). Calculate lipid ratios reflecting lipid metabolism, including TG/HDL-C, TC/HDL-C, LDL-C/HDL-C, ApoA/ApoB. Body Mass Index (BMI) was calculated as weight (kg) divided by height squared (m^2^).

### 2.4 Coronary angiography

All study participants underwent coronary angiography, and the Gensini score for each patient was independently assessed by at least two interventional cardiologists, to obtain the average score. Coronary artery stenosis severity was quantitatively evaluated according to the American College of Cardiology and American Heart Association guidelines for coronary angiography. The scoring criteria for coronary artery stenosis and lesion location were as follows: ≤25% stenosis was scored as 1 point; 26%–50% stenosis as 2 points; 51%–75% stenosis as 4 points; 76%–90% stenosis as 8 points; 91%–99% stenosis as 16 points; and total occlusion as 32 points. Different lesion locations had varying importance in coronary circulation, necessitating multiplication by different location coefficients (e.g., if the lesion was in the left main coronary artery, the result was multiplied by 5; for the proximal left anterior descending artery, it was multiplied by 2.5; for the mid segment, by 1.5; and for the distal segment, by 1; the proximal left circumflex artery by 2.5; the mid and distal segments of the circumflex artery, first diagonal branch, obtuse marginal branch, right coronary artery, and posterior descending artery by 1; the second diagonal branch and posterior lateral branch by 0.5, etc.) (24).

The Gensini score is the sum of the products of the severity of stenosis for each coronary artery and the coefficients determined by lesion location, with a higher Gensini score indicating more severe coronary artery stenosis. The patients were divided into two groups based on the median Gensini score: a mild stenosis group with 217 cases (Gensini score <36) and a moderate-to-severe stenosis group with 235 cases (Gensini score ≥36). “Multivessel lesions” was defined as at least two vessels with a diameter of 2.25–5.75 mm had ≥70% stenosis, and the culprit lesion could be clearly identified (25).

### 2.5 Statistical analysis

The participants were divided into two groups based on the median Gensini score, and the baseline characteristics of the two groups were compared. For descriptive statistics, continuous variables with a normal distribution were presented as mean ± standard deviation, and intergroup comparisons were performed using Welch's *t*-test. Continuous variables with a non-normal distribution were expressed as median (*P_25_*, *P_75_*), and intergroup comparisons were conducted using the Mann–Whitney *U* test. Categorical variables were presented as counts (n) or percentages (%), and intergroup comparisons were performed using the chi-square test. Missing data (excluding lipid and angiographic parameters with a total missing rate not exceeding 10%) were collected using multiple imputation methods. All statistical analyses were performed using R statistical software (Version 9 4.2.2, http://www.R-project.org, The R Foundation). FreeStatistics is a software package provides intuitive interfaces for most common analyses and data visualization. It uses R as the underlying statistical engine, and the graphical user interface (GUI) is written in Python. *P* < 0.05 was considered statistically significant.

We employed multivariate logistic regression analysis to accurately assess the independent association between moderate-to-severe coronary artery stenosis (Gensini score ≥36) and RC and lipid ratios, both as continuous and categorical variables, by controlling for multiple confounding factors. In Model 1, we adjusted for basic demographic variables and lifestyle factors including sex, age, BMI, history of hypertension, history of diabetes, smoking, and alcohol intake. Model 2 was further adjusted for a history of hyperlipidemia to control for the effect of lipid abnormalities on the results. Model 3 expanded on Model 2 by additionally adjusting for the multivessel lesions. Moreover, after adjusting for the same confounding factors, we explored the effect of inconsistency between RC and non-HDL-C on the risk of severe coronary stenosis, based on the curve-fitting inflection points and 75th percentiles. Finally, to evaluate the predictive value of RC and lipid ratios for coronary stenosis severity, we performed receiver operating characteristic (ROC) curve analysis. The predictive performance of these indicators was quantified by calculating the area under the curve (AUC). We also constructed a predictive model incorporating four lipid ratios, and subsequently developed a novel combined predictive model by integrating RC with other lipid ratios to enhance predictive accuracy.

After each subject was enrolled, the researchers filled out a self-designed baseline survey form for CHD, recording detailed information, such as the patient's name, sex, age, relevant tests, and examination data. All data were sourced from patients’ original hospital records. To ensure the quality of the study, all researchers involved in data collection and evaluation were required to undergo uniform training to standardize the completion of baseline survey forms and the evaluation process of the Gensini scores.

## 3 Results

### 3.1 Baseline characteristics comparison

This study involved an average patient age of 60.5 ± 10.4 years, with men constituting 73.23% of the cohort. A history of smoking was noted in 40.71% of the patients, and 11.06% (50 patients) reported alcohol intake. Comorbid diabetes was present in 25.22% (114 cases), hyperlipidemia in 16.59% (75 cases), and multivessel coronary artery lesions in 80.31% (363 cases). There were significant differences between the mild and moderate-to-severe stenosis groups in terms of age, prevalence of hyperlipidemia, and multivessel involvement (*P* < 0.05). Lipid parameters such as TG, TC, LDL-C, ApoB, non-HDL-C, and RC, as well as lipid ratios (TG/HDL-C, TC/HDL-C, LDL-C/HDL-C, and ApoB/ApoA) were notably higher in the group with higher Gensini scores (*P* < 0.05), while HDL-C was significantly lower (*P* < 0.05) (Table 1).

**Table 1:** Baseline characteristics according to gensini subgroups.

Characteristic	All participants (*n* = 452)	Gensini score <36 (*n* = 217)	Gensini score ≥ 36 (*n* = 235)	*p*-value
Sex, *n* (%)				0.817^1^
Male	331 (73.23)	160 (73.73)	171 (72.77)	
Female	121 (26.77)	57 (26.27)	64 (27.23)	
Age, years	60 (52, 67)	62 (54, 68)	58 (51, 66)	0.011^2^
BMI, kg/m^2^	25.01 ± 2.85	25.03 ± 2.68	25.00 ± 3.01	0.908^3^
Hypertension, *n* (%)				0.419^1^
No	154 (34.07)	78 (35.94)	76 (32.34)	
Yes	298 (65.93)	139 (64.06)	159 (67.66)	
Diabetes, *n* (%)				0.094^1^
No	338 (74.78)	170 (78.34)	168 (71.49)	
Yes	114 (25.22)	47 (21.66)	67 (28.51)	
Hyperlipidemia, *n* (%)				0.023^1^
No	377 (83.41)	190 (87.56)	187 (79.57)	
Yes	75 (16.59)	27 (12.44)	48 (20.43)	
Smoking, former or current smokers *n* (%)				0.160^1^
No	268 (59.29)	136 (62.67)	132 (56.17)	
Yes	184 (40.71)	81 (37.33)	103 (43.83)	
Alcohol intake, *n* (%)				0.999^1^
No	402 (88.94)	193 (88.94)	209 (88.94)	
Yes	50 (11.06)	24 (11.06)	26 (11.06)	
Multibranch lesions, *n* (%)				<0.001^1^
No	89 (19.69)	61 (28.11)	28 (11.91)	
Yes	363 (80.31)	156 (71.89)	207 (88.09)	
TG, mmol/L	1.73 (1.26, 2.47)	1.52 (1.10, 2.06)	1.95 (1.47, 2.81)	<0.001^2^
TC, mmol/L	4.50 (3.85, 5.21)	4.24 (3.68, 4.98)	4.66 (4.05, 5.36)	<0.001^2^
HDL-C, mmol/L	1.00 (0.85, 1.20)	1.03 (0.90, 1.25)	0.97 (0.82, 1.16)	0.001^2^
LDL-C, mmol/L	2.90 (2.35, 3.51)	2.71 (2.24, 3.30)	3.04 (2.47, 3.61)	<0.001^2^
ApoA, g/L	1.20 (1.07, 1.35)	1.22 (1.09, 1.39)	1.18 (1.05, 1.34)	0.067^2^
ApoB, g/L	1.01 (0.85, 1.20)	0.95 (0.80, 1.12)	1.09 (0.88, 1.23)	<0.001^2^
non-HDL-C, mmol/L	3.45 (2.82, 4.12)	3.12 (2.60, 3.97)	3.72 (3.07, 4.43)	<0.001^2^
RC, mmol/L	0.48 (0.31, 0.71)	0.36 (0.24, 0.56)	0.58 (0.43, 0.81)	<0.001^2^
TG/HDL-C	1.71 (1.13, 2.69)	1.41 (0.94, 2.18)	1.94 (1.40, 3.19)	<0.001^2^
TC/HDL-C	4.40 (3.64, 5.30)	4.01 (3.34, 4.83)	4.78 (3.89, 5.71)	<0.001^2^
LDL-C/HDL-C	2.91 (2.25, 3.58)	2.65 (2.02, 3.26)	3.08 (2.41, 3.82)	<0.001^2^
ApoB/ApoA	0.85 (0.69, 1.03)	0.79 (0.65, 0.94)	0.91 (0.75, 1.09)	<0.001^2^
FPG, mmol/L	5.70 (5.10, 7.20)	5.60 (4.90, 7.00)	5.90 (5.20, 7.45)	0.018^2^
CK, U/L	144 (83, 458)	129 (81, 432)	153 (87, 471)	0.331^2^
CK-MB, U/L	20 (14, 47)	20 (13, 45)	21 (14, 49)	0.353^2^
cTnI, ng/ml	0.2 (0.0, 1.6)	0.2 (0.0, 1.4)	0.2 (0.0, 1.7)	0.268^2^
NT-proBNP, pg/ml	313 (101, 999)	201 (62, 646)	410 (146, 1,122)	<0.001^2^
LVEF (%)	61 (56, 64)	61 (58, 64)	60 (56, 63)	<0.050^2^
Gensini Score	36 (26, 50)	25 (21, 30)	50 (43, 56)	<0.001^2^

BMI, body mass index; TG, triglyceride; TC, total cholesterol; HDL-C, high-density lipoprotein cholesterol; LDL-C, low-density lipoprotein cholesterol; ApoA, apolipoprotein A; ApoB, apolipoprotein B; non-HDL-C, non-high-density lipoprotein cholesterol; RC, remnant cholesterol; FPG, fasting plasma glucose; CK, creatine kinase; CK-MB, creatine kinase isoenzyme; cTnI, cardiac troponin I; NT-proBNP, N-terminal precursor of brain natriuretic peptide; LVEF, left ventricular ejection fraction. *P* < 0.05 was considered statistically significant.

Values were presented as *n* (%), Median (IQR) or Mean ± SD.

^1^ Chi-squared test.

^2^ Mann–Whitney *U* test.

^3^ Welch's *t*-test; To convert triglyceride values from mg/dl to mmol/L, multiply by 0.0113; to convert cholesterol values from mg/dl to mmol/L, multiply by 0.0259; to convert glucose values from mg/dl to mmol/L, multiply by 0.0556.

### 3.2 Rc, lipid ratios and moderate to severe coronary artery stenosis

**Figure 2:**
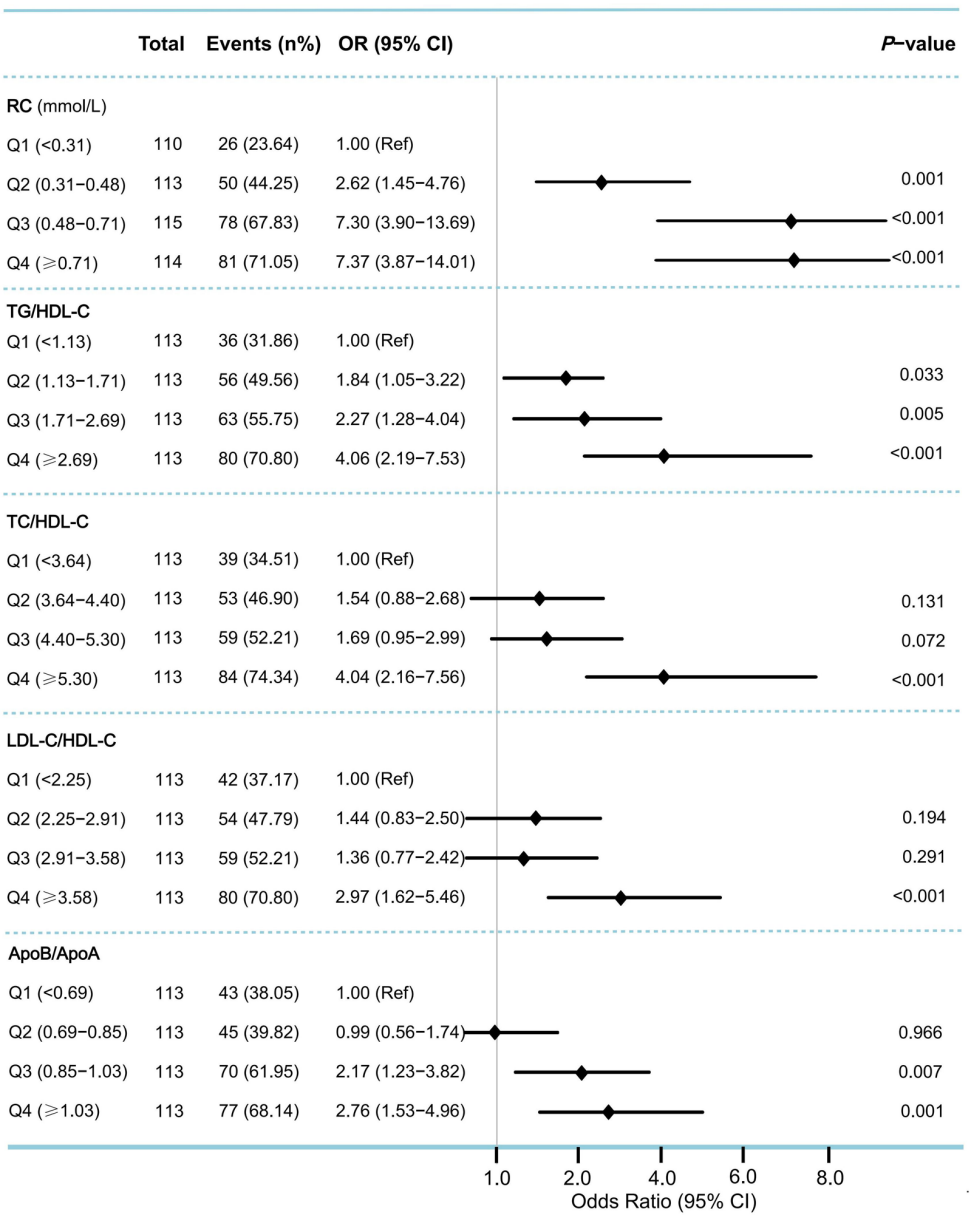
Odds ratios (95% CI) for moderate to severe coronary artery stenosis according to quartiles of remnant cholesterol and lipid ratios. To assess the risk of moderate to severe coronary stenosis associated with the baseline remnant cholesterol and lipid ratios, we calculated ORs for the second, third, and fourth quartiles (compared with the first quartile) of levels of remnant cholesterol and various lipid ratios. Moderate to severe coronary stenosis was defined as Gensini score ≥36. ORs were adjusted for the same covariates as Model 3 in Table 2. OR, odds ratio; CI, confidence interval; Ref, reference.

**Figure 3:**
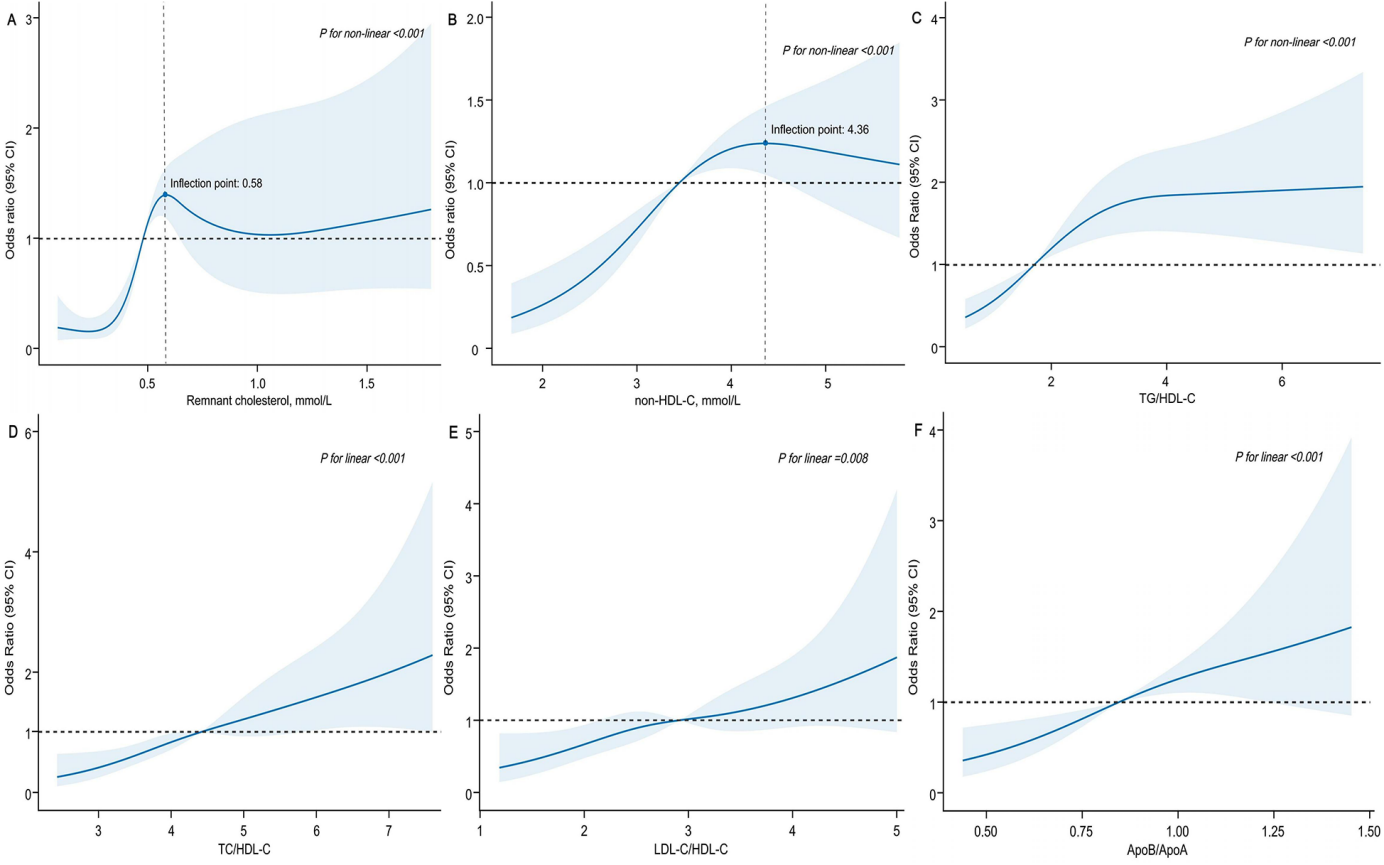
Association of remnant cholesterol, non-HDL-C and lipid ratios with moderate to severe coronary artery stenosis among patients with CHD. *X*-axis represented remnant cholesterol **(A)**, non-HDL-C **(B)** and various lipid ratios **(C–F)**, respectively. *Y*-axis represented the OR to present moderate to severe coronary stenosis for any value of RC (non-HDL-C and various lipid ratios) compared to individuals with reference value (50th percentile) of RC (non-HDL-C and various lipid ratios). ORs were adjusted for the same covariates as Model 3 in Table 2. Abbreviations as in Table 1.

In the multivariable logistic regression analysis, moderate to severe coronary artery stenosis (Gensini score ≥36) was used as the dependent variable. After adjusting for confounding factors, including age, sex, BMI, smoking, alcohol intake, medical history (such as hypertension, diabetes, and hyperlipidemia), and multivessel lesions, the results showed that RC and lipid ratios (TG/HDL-C, TC/HDL-C, LDL-C/HDL-C, and ApoB/ApoA) were independent risk factors for moderate-to-severe coronary artery stenosis. The strongest predictors were RC [odds ratio [OR] 5.44, 95% confidence interval [CI]: 2.65–11.15, *P* < 0.001], ApoB/ApoA (OR: 5.00, 95% CI: 2.17–11.51, *P* < 0.001), and TC/HDL-C (OR: 1.49, 95% CI: 1.24–1.79, *P* < 0.001) (Table 2). Grouping RC and lipid ratios by quartiles as categorical variables showed an increased risk of moderate to severe coronary artery lesions from Q1 to Q4, with the Q4 group for RC having 7.37 times the risk of the Q1 group in Model 3 (*P* < 0.001) (Figure 2). Similar results were observed in the unadjusted model, Model 1, and Model 2, with consistent and robust associations. Detailed results are presented in Supplementary Table 1. Moreover, possible nonlinear relationships between changes in RC, non-HDL-C, various lipid ratios, and moderate to severe coronary stenosis were examined using restricted cubic spline regression. The results showed a nonlinear relationship between RC, non-HDL-C, TG/HDL-C, and moderate-to-severe coronary stenosis in patients with CHD (*P for non-linear* < 0.001), whereas other lipid ratios showed a nearly linear relationship with moderate to severe coronary stenosis events (*P for linear* < 0.05) (Figure 3).

**Table 2:** Logistic regression models (95% CI) for moderate to severe coronary artery stenosis based on remnant cholesterol and lipid ratios (continuous variables) in CHD patients.

Variables	Model 1, OR (95% CI)	Model 2, OR (95% CI)	Model 3, OR (95% CI)	*p*
RC, mmol/L	5.76 (2.91, 11.41)	5.48 (2.71, 11.08)	5.44 (2.65, 11.15)	<0.001
TG/HDL-C	1.25 (1.09, 1.43)	1.23 (1.07, 1.40)	1.22 (1.06, 1.40)	0.006
TC/HDL-C	1.57 (1.32, 1.86)	1.55 (1.29, 1.85)	1.49 (1.24, 1.79)	<0.001
LDL-C/HDL-C	1.59 (1.28, 1.98)	1.55 (1.24, 1.94)	1.47 (1.17, 1.84)	0.001
ApoB/ApoA	6.29 (2.78, 14.24)	5.82 (2.55, 13.28)	5.00 (2.17, 11.51)	<0.001

Moderate to severe coronary artery stenosis was defined as Gensini score ≥36.

Model 1: adjusted for sex, age, BMI, hypertension, diabetes, smoking and alcohol intake.

Model 2: Model 1 + adjusted for hyperlipidemia.

Model 3: Model 2 + adjusted for multibranch lesions.

TG/HDL-C, the ratio of triglycerides divided by high-density lipoprotein cholesterol. The calculation methods for other lipid ratios were similar; OR, odds ratio; CI, confidence interval. Other abbreviations as in Table 1.

### 3.3 Inconsistency between RC and non-HDL-C and moderate to severe coronary artery stenosis

When evaluating the risk of moderate to severe coronary artery stenosis (Gensini score ≥36) as the dependent variable, and consistency or inconsistency between RC and non-HDL-C levels as the independent variable, compared with the consistent low RC group, results showed that the consistent high RC group had the greatest risk (OR: 4.32, 95% CI: 2.15–8.66, *P* < 0.001), similar to that of the inconsistent high RC group (OR: 2.72 95% CI: 1.64–4.52, *P* < 0.001). The inconsistent low RC group had the smallest risk (OR: 0.80, 95% CI: 0.33–1.95, *P* > 0.05) (Model 3). In other words, irrespective of the non-HDL-C levels, an increase in RC significantly increased the risk of moderate to severe coronary narrowing. When using the third quartiles of RC and non-HDL-C for the inconsistency analysis, similar results were observed in the high RC/low non-HDL-C group without adjusting for covariates or in Model 1. However, after further adjusting for the history of hyperlipidemia, the results showed *P*-values exceeded 0.05 in both Models 2 and 3, indicating that this covariate had a significant effect on the inconsistency analysis based on the quartile grouping (Table 3).

**Table 3:** Odds ratios (95% CI) for moderate to severe coronary artery stenosis across non-HDL-C vs. remnant cholesterol concordant/discordant groups by curve fitting inflection points (or the third quartiles) of non-HDL-C and remnant cholesterol.

Models and case distribution	non-HDL-C < 4.36 RC < 0.58	non-HDL-C < 4.36 RC ≥ 0.58	*P*	non-HDL-C ≥ 4.36 RC < 0.58	*P*	non-HDL-C ≥ 4.36 RC ≥ 0.58	*P*-value
Cases, *n* (%)	104 (40.6)	69 (66.3)		10 (37.0)		52 (80.0)	
Unadjusted model	(Ref)	2.88 (1.79, 4.64)	<0.001	0.86 (0.38, 1.95)	0.718	5.85 (3.03, 11.28)	<0.001
Model 1	(Ref)	2.74 (1.68, 4.45)	<0.001	0.86 (0.37, 1.98)	0.718	5.31 (2.72, 10.39)	<0.001
Model 2	(Ref)	2.63 (1.6, 4.32)	<0.001	0.78 (0.32, 1.86)	0.568	5.00 (2.51, 9.93)	<0.001
Model 3	(Ref)	2.72 (1.64, 4.52)	<0.001	0.80 (0.33, 1.95)	0.623	4.32 (2.15, 8.66)	<0.001
Models and case distribution	non-HDL-C < 4.12 RC < 0.71	non-HDL-C < 4.12 RC ≥ 0.71	*P*	non-HDL-C ≥ 4.12 RC < 0.71	*P*	non-HDL-C ≥ 4.12 RC ≥ 0.71	*P*-value
Cases, *n* (%)	127 (45.2)	34 (61.8)		27 (47.4)		47 (79.7)	
Unadjusted model	(Ref)	1.96 (1.09, 3.55)	0.026	1.09 (0.62, 1.93)	0.764	4.75 (2.42, 9.34)	<0.001
Model 1	(Ref)	1.85 (1.01, 3.38)	0.047	1.08 (0.6, 1.94)	0.79	4.29 (2.16, 8.54)	<0.001
Model 2	(Ref)	1.69 (0.91, 3.16)	0.098	0.97 (0.53, 1.79)	0.922	3.98 (1.98, 8.0)	<0.001
Model 3	(Ref)	1.72 (0.91, 3.26)	0.093	0.88 (0.47, 1.64)	0.688	3.42 (1.69, 6.94)	0.001

Unadjusted model: no covariates applied. The adjusted covariates in the models were consistent with Table 2. The inflection points from curve fitting for non-HDL-C and RC were 4.36 mmol/L and 0.58 mmol/L, respectively, while their third percentiles were 4.12 mmol/L and 0.71 mmol/L, respectively. non-HDL-C, non-high-density lipoprotein cholesterol; RC, remnant cholesterol. Other abbreviations as in Table 1.

### 3.4 Predictive value of RC, lipid ratios, and combined predictive factors on moderate to severe coronary artery stenosis

**Figure 4:**
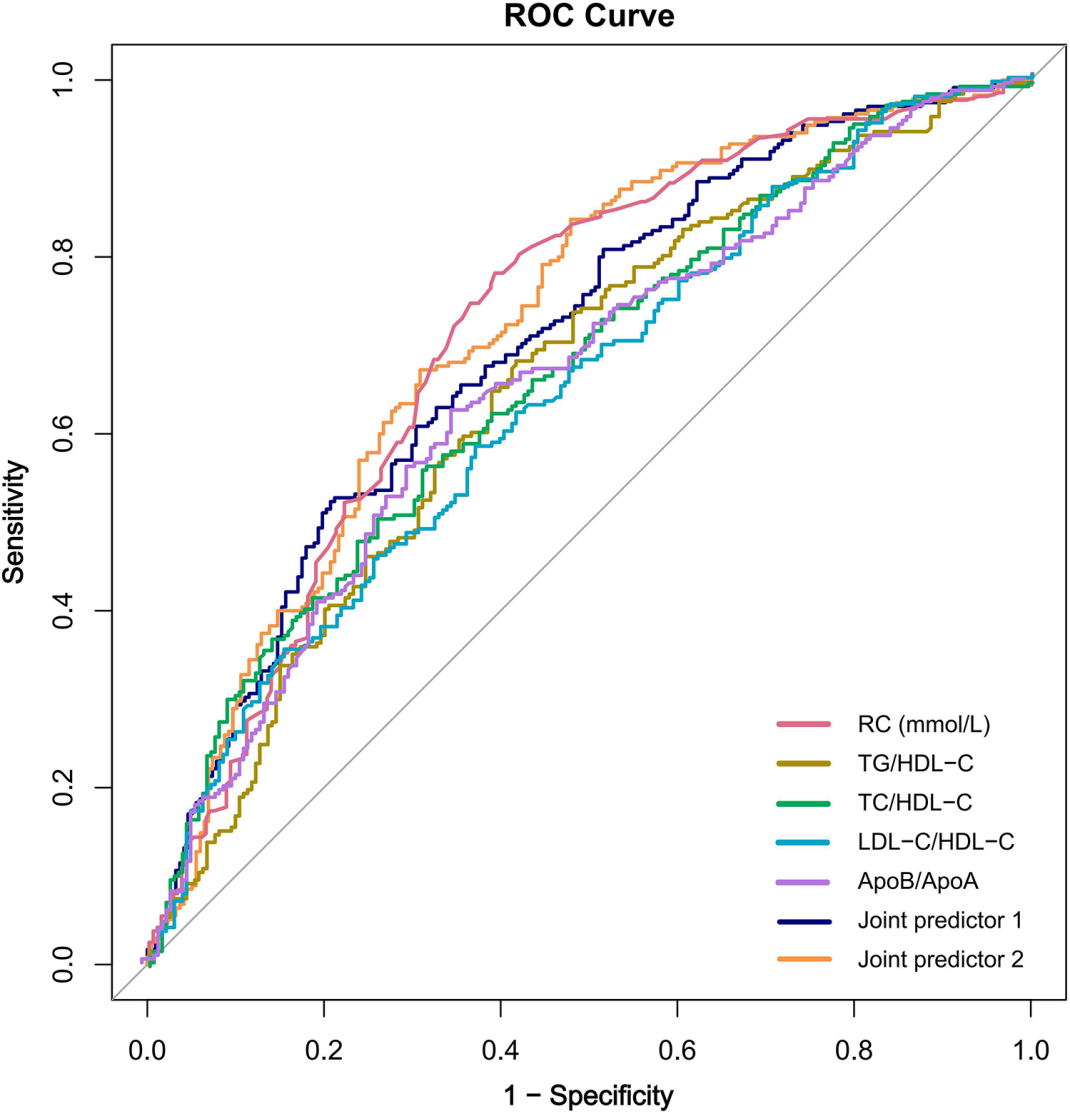
The receiver operating characteristic curve of remnant cholesterol, lipid ratios and joint predictors. Joint predictor 1: TG/HDL-C + TC/HDL-C + LDL-C/HDL-C + ApoB/ApoA; Joint predictor 2: RC + Joint predictor 1; RC, remnant cholesterol; ROC, receiver operating characteristic.

ROC curve analysis was used to evaluate the predictive value of RC and lipid ratios for high Gensini scores. The top three predictors based on the AUC were RC, TC/HDL-C, and TG/HDL-C, with RC having the highest AUC of 0.715 (sensitivity 77.4%, specificity 60.8%) (Table 4). Adding RC to a predictive model composed of lipid ratios (Combined Predictive Factor 1) to create Combined Predictive Factor 2 showed that except for the comparison with the AUC of RC, significant differences were observed with other indicators (*P* < 0.05), indicating that RC significantly enhanced the predictive power of the lipid ratio assessment model (Figure 4).

**Table 4:** Comparison of area under ROC curve of remnant cholesterol, lipid ratios and joint predictors.

Variables	AUC (95% CI)	Cutoff	Sensitivity	Specificity	*P*
RC, mmo/L	0.715 (0.667, 0.763)	0.420	77.4%	60.8%	> 0.05
TG/HDL-C	0.661 (0.611, 0.711)	1.609	68.5%	58.5%	< 0.01
TC/HDL-C	0.671 (0.622, 0.720)	4.653	56.2%	69.1%	< 0.01
LDL-C/HDL-C	0.640 (0.589, 0.690)	2.962	57.9%	63.1%	< 0.01
ApoB/ApoA	0.649 (0.599, 0.700)	0.850	62.6%	65.0%	< 0.01
Joint predictor 1	0.703 (0.656, 0.751)	—	52.3%	79.3%	0.041
Joint predictor 2	0.723 (0.675, 0.770)	—	67.2%	69.1%	Ref

Joint predictor 1:TG/HDL-C + TC/HDL-C + LDL-C/HDL-C + ApoB/ApoA; Joint predictor 2:RC + Joint predictor 1.

ROC, receiver operating characteristic; AUC, area under the curve. Other abbreviations as in Table 1.

## 4 Discussion

This study found that RC, along with various lipid ratios (TG/HDL-C, TC/HDL-C, LDL-C/HDL-C, and ApoB/ApoA) played a critical role in assessing the severity of coronary artery stenosis in patients with CHD. Notably, RC demonstrated robust predictive power for the severity of coronary stenosis, independent of traditional risk factors such as age, sex, and non-HDL-C levels. Moreover, the predictive efficacy of RC was significantly higher than that of traditional lipid indices and ratios when used alone. When combined with other lipid ratios, RC further enhanced the predictive value of moderate to severe coronary artery lesions.

It is widely accepted that lipid deposition caused by lipid metabolism disorder is the most direct pathogenic factor of atherosclerosis. Studies have indicated that an elevated RC significantly increases the risk of cardiovascular events, and paying attention to the detection and assessment of RC could help identify potential CVDs that LDL-C cannot predict. Analysis of the PREDIMED trial showed that for every 0.26 mmol/L increase in RC, the risk of MACEs rose by 21%, and subjects with RC  ≥  0.78 mmol/L (75th percentile of the cohort) had a higher risk of MACEs regardless of whether LDL-C was maintained within the optimal range (≤2.59 mmol/L). RC levels, rather than LDL-C or HDL-C levels, have been demonstrated to correlate with MACEs independent of cardiovascular risk factors and lipid-lowering therapy (26). Furthermore, a large Mendelian randomization study showed that an elevated RC increased the risks of CHD, myocardial infarction (MI), and stroke 1.51, 1.57, and 1.23 times, respectively. This study was the first to demonstrate a direct causal relationship between high RC levels and CVD risk in a large population, emphasizing the effect of RC on CHD and MI independent of LDL-C levels (11). Matsuo et al. indicated that high levels of RC in patients led to easier accumulation of cholesterol in the arterial wall, which could induce AS and form thin-cap fibroatheroma plaques. These plaques had a higher risk of rupture and significantly increased risk of recurrent cardiovascular events in patients with CHD (27). In a prospective study involving 6,544 individuals without atherosclerotic cardiovascular disease (ASCVD), RC levels were closely associated with the severity of coronary artery calcification, a relationship that remained significant even in patients with well-controlled LDL-C levels (28).

Some results of the present study are consistent with those of previous studies, which showed that CHD patients with moderate-to-severe coronary artery stenosis had significantly higher RC levels than those with mild stenosis. After adjusting for confounding factors in the multiple regression model, we found that the RC, TC, TG, ApoB, and lipid ratios were independent risk factors for moderate to severe coronary lesions in patients with CHD. Notably, the OR value for RC reached 5.44 (Model 3), indicating the strongest association among the various lipid indicators and demonstrating the critical role of RC in the development of severe coronary stenosis in patients with CHD. ROC curve analysis provided more intuitive evidence; the AUC for RC was 0.715, surpassing the predictive efficacy of single lipid ratios and even combined predictive factor 1, further validating the important predictive value of RC for a high Gensini score.

Recent studies have suggested that elevated fasting RC levels are positively correlated with the average carotid intima-media thickness (cIMT) and maximum cIMT in patients with IS, even among those with optimal LDL-C levels, indicating that RC might serve as a potential indicator for risk stratification of carotid atherosclerotic stenosis (29). In a multivariate Mendelian randomization analysis, RC was found to be closely and independently associated with CHD after adjusting for ApoB levels. Similarly, in multivariable models, RC and LDL-C showed independent associations with CHD, with ORs of 2.59 and 1.37 for each 1 mmol/L increase in cholesterol, respectively (30). Compared with LDL, TRL/RC appears to be more prominent in promoting AS on a per-particle basis. This study also indicated that CHD patients with a high RC had a higher incidence of severe coronary artery stenosis than those with a lower RC. The exact mechanisms remain unclear, but RC may be one of the factors that trigger AS formation. First, the larger quantity and volume of RC compared with LDL allows it to carry more cholesterol and be taken up by macrophages without requiring oxidative modification, leading to the formation of foam cells and AS (16). Thus, RC may have a stronger atherogenic effect than LDL-C. Second, RC can effectively activate endothelial cells and monocytes, triggering a low-grade inflammatory response that exacerbates arterial intimal damage and increases plaque instability (31). Furthermore, RC promotes oxidative stress and the production of lipid peroxidation products, further aggravating endothelial cell injury and dysfunction and ultimately facilitating thrombosis. During this process, a vicious cycle of inflammation and thrombosis accelerates AS formation and progression (32). In summary, when lipid metabolism is imbalanced, elevated levels of RC can lead to its infiltration into the arterial wall, causing cholesterol deposition beneath the endothelium and accelerating the formation of foam cells and lipid plaques, while provoking a low-grade inflammatory response. These processes significantly increase the risk of AS. Therefore, RC may theoretically provide a better predictor of the severity of coronary atherosclerotic lesions. Further studies are required to elucidate the specific mechanisms underlying the association between RC and AS.

Our study also found that lipid ratios such as the ApoB/ApoA-1 ratio (BAR) were closely associated with the degree of coronary artery stenosis and might have a potential predictive value for CHD severity. Several observational and genetic studies have consistently indicated that lipid ratios were more accurate than traditional lipid markers such as LDL-C in reflecting the risk of cardiovascular events. A prospective cohort study revealed that in patients with CHD undergoing coronary intervention, BAR performed better in predicting coronary occlusion than a single lipid marker or the non-HDL-C/HDL-C ratio (33). Additionally, a Mendelian randomization (MR) study utilizing summary data from a genome-wide association study (GWAS) of the ApoB/ApoA1 ratio (BAR), Lp(a), and TG in a European population confirmed a causal relationship between BAR and CHD, emphasizing that BAR was an independent risk factor for CHD (34). In this study, among patients with CHD who had not previously used lipid-lowering drugs, we observed that for every unit increase in baseline fasting BAR, the risk of moderate to severe coronary lesions increased by 5.00 times (Model 3), which was similar to the OR value of RC (OR = 5.44). Both are independent risk factors for moderate-to-severe coronary stenosis. However, in the ROC curve analysis, there was a significant difference in the AUC for high Gensini score between the two (0.715 vs. 0.649, *P* < 0.01). Compared with RC, BAR did not show the expected advantage in assessing severe coronary lesions. Nevertheless, the above evidence supports the key role of BAR in the prevention and treatment of CVD, and warrants further exploration of its application value in a broader population of patients with CHD.

A meta-analysis of 12 high-quality clinical studies found a positive correlation between LDL-C/HDL-C ratio (LHR) and CHD, suggesting that LHR may serve as a potential indicator for assessing CHD risk (35). Lo et al. investigated the effect of the TG/HDL-C ratio on all-cause and cardiovascular mortality in the general population. The results indicated a nonlinear relationship between TC/HDL-C ratio and all-cause mortality, where both elevated and reduced TC/HDL-C ratios increased the risk of all-cause mortality. However, in terms of cardiovascular mortality, individuals with a TC/HDL-C ratio >4.22 showed a significantly increased risk of cardiovascular death (36). Insulin resistance plays a crucial role in the development of AS (37, 38). The significance of the TG/HDL-C ratio, a key surrogate marker of insulin resistance, was demonstrated in a recent prospective cohort study. After adjusting for various CVD risk factors, patients in the highest quartile of the TG/HDL-C ratio had a 1.29-fold increase in overall CVD risk compared with those in the lowest quartile, highlighting the potential value of the TG/HDL-C ratio in assessing CVD risk (39). Building on these studies, which reflected the relationship between lipid ratios and CVD risk, our study further combined RC with various lipid ratios to create a joint predictive model. The results indicated that the combined use of RC and lipid ratios significantly improved the predictive capability of the severity of coronary artery stenosis. As an independent marker of residual lipid risk, RC supplements traditional lipid indicators and provides additional information regarding AS burden. Additionally, we chose composite lipid indicators—lipid ratios—which comprehensively reflected the dual mechanisms of AS, the dynamic balance between “causative factors” (LDL-C, ApoB) and “protective factors” (HDL-C, ApoA). Based on this characteristic, we had reason to speculate that the association between lipid ratios and the severity of coronary lesions might surpass that of any single lipid component. By establishing a joint predictive factor using logistic regression models with RC and other lipid ratios and plotting the ROC curve, the AUC of the joint predictive factor was 0.723, sensitivity was 67.2%, and specificity was 69.1%, exceeding the predictive efficacy of the individual lipid ratios.

Although the accuracy of calculated RC is limited by the precision of TG, HDL-C, and LDL-C measurements, as well as the presence of small amounts of chylomicrons in fasting plasma, we tend to adopt a more efficient calculation method, given the high cost of direct RC measurement with current technology. However, the calculated clinical value of RC is much more than the simple difference between TC and LDL-C or HDL-C in the formula. A prospective cohort study based on the China-PAR project found that elevated inconsistency in RC, rather than elevated LDL-C, was an independent factor for increasing the risk of stroke and IS, but was not related to the risk of hemorrhagic stroke (40). A study by Quispe et al. analyzed the correlation between the inconsistency between RC and LDL-C and the risk of ASCVD, and found that the inconsistent high RC/low LDL-C group, rather than the low RC/high LDL-C group, had a significantly increased ASCVD risk (HR: 1.21, 95% CI: 1.08–1.34) compared with the consistently low group, and similar results were also shown when using different clinical cut-off values for analysis (41). In the RC calculation formula, there is some overlap with non-HDL-C. Therefore, this study also evaluated whether the inconsistency between RC and non-HDL-C was related to the severity of coronary artery stenosis in patients with CHD. When grouping by inflection points in curve fitting, results showed that patients with high RC/low non-HDL-C had a significantly higher risk of severe coronary artery lesions than those with consistently low RC and non-HDL-C levels (OR: 2.72, 95% CI: 1.64–4.52, *P* *<* 0.001). However, this study also indicated that compared with patients with consistent low levels of RC and non-HDL-C, the inconsistency of low RC/high non-HDL-C did not show a significant association with moderate to severe coronary stenosis (OR: 0.8, 95% CI: 0.33–1.95, *P* > 0.05). This observation suggests that RC may have higher accuracy than non-HDL-C in predicting the severity of CHD. Whether non-HDL-C is at an ideal level, individuals with high RC levels still face the risk of developing CHD with severe coronary lesions. This residual risk may be closely related to the inconsistencies in lipid levels.

This study focused on exploring the relationship between calculated RC and the severity of coronary artery lesions in patients. However, it is important to emphasize that the accuracy of RC calculation largely depends on the reliability of LDL-C measurements. In clinical practice and many large-scale studies, LDL-C is commonly estimated using the Friedewald formula (LDL-C = TC − HDL-C − TG/2.2, mmol/L), which assumes that VLDL-C accounts for approximately one-fifth of TG. However, this assumption may lead to significant errors in individuals with high TG levels (>1.7 mmol/L) and low LDL-C levels (<1.8 mmol/L), thereby affecting the accuracy of RC estimation (42). Although the Martin-Hopkins formula provides a more accurate estimation of LDL-C compared to the Friedewald formula in patients with LDL-C levels <1.8 mmol/L and TG levels between 1.7 and 4.5 mmol/L, both calculation methods have limitations when TG levels are markedly elevated (>4.5 mmol/L) (43, 44). To circumvent the potential errors introduced by these calculation formulas, our study employed direct LDL-C measurement, thereby improving the accuracy of RC calculation and the reliability of our findings to some extent, which was critical for objectively evaluating RC as an independent predictor of coronary artery stenosis severity in CHD. Nevertheless, direct LDL-C measurement remains costly, limiting its widespread implementation in large-scale clinical practice. Hence, calculation methods like the Martin-Hopkins formula still offer distinct advantages in terms of reducing personnel and financial costs.

### 4.1 Strengths and limitations

Our study was the first to evaluate the potential association between fasting RC levels and the severity of coronary artery stenosis in patients with CHD and innovatively introduced a series of lipid ratios to construct a comprehensive assessment model. ROC curve analysis revealed that this combined predictor significantly outperformed other single lipid ratios in terms of predictive efficacy, particularly with the addition of RC, which markedly enhanced the predictive ability of the lipid ratio model. The combined predictive factors selected in this study demonstrated an optimal predictive value for the accurate assessment of the risk of coronary artery lesions. Furthermore, our study confirmed that inconsistency in lipid levels (RC and non-HDL-C) was associated with the severity of coronary lesions in patients with CHD, analyzing potential factors leading to severe coronary stenosis events from another perspective and providing additional reference information for identifying high-risk patients.

This study has several limitations. First, a small number of enrolled patients had a history of statin use, and we were unable to accurately collect detailed information regarding the duration and dosage of statin therapy, which may have affected the predictive efficacy of certain lipid parameters. Second, the study was based solely on baseline data, and the dynamic impact of changes in RC and lipid ratios over time on the severity of coronary artery stenosis was not evaluated. Additionally, as a single-center retrospective study with a relatively limited sample size, the statistical significance of the results may not fully reflect their clinical relevance. To minimize this limitation, we employed rigorous statistical methods, including multiple model analyses and ROC curve analyses. The results remained consistent across different models, confirming the robustness of our findings. Looking ahead, future multi-center, large-scale prospective studies are warranted to further validate our findings and comprehensively assess the roles of RC and lipid ratios in the long-term prognosis and clinical risk assessment of CHD.

## 5 Conclusions

We found that RC has the potential to become an independent predictive marker for moderate to severe coronary artery narrowing in patients with CHD beyond traditional lipid indices. When used in conjunction with other lipid ratios, RC significantly enhanced the predictive value of lipid ratio models. These findings support the integration of RC and lipid ratios into a comprehensive risk assessment framework for CHD. Future prospective studies are needed to verify our findings and explore the effect of lowering RC on the prognosis of patients with CHD.

## Data Availability

The original contributions presented in the study are included in the article/Supplementary Material, further inquiries can be directed to the corresponding authors.
