# Supplementary material for: Remnant cholesterol, lipid ratios, and the severity of coronary artery lesions: a retrospective cohort study in patients with coronary heart disease
Source: Front Cardiovasc Med. 2025 Mar 10;12:1516326. doi: 10.3389/fcvm.2025.1516326 (PMC11930836; doi:10.3389/fcvm.2025.1516326)
Supplement: Supplementary file 1 [file Table1.doc]

**Supplementary Table 1 Odds ratios (95% CI) for moderate to severe coronary artery stenosis according to quartiles of remnant cholesterol and lipid ratios in unadjusted model, model 1, and model 2**

| **Variables** | **Events (%)** | **Unadjusted model** | | **Model 1** | | **Model 2** | |
| --- | --- | --- | --- | --- | --- | --- | --- |
|  |  | **OR (95%CI)** | ***P*** | **OR (95%CI)** | ***P*** | **OR (95%CI)** | ***P*** |
| **RC, mmol/L** |  |  |  |  |  |  |  |
| Per SD increase | — | 2.06 (1.7, 2.48) | 0.001 | 2.01 (1.66, 2.43) | < 0.001 | 2.01 (1.64, 2.45) | < 0.001 |
| Q1: < 0.31 | 26 (23.64) | 1.00 (Ref) | — | 1.00 (Ref) | — | 1.00 (Ref) | — |
| Q2: 0.31≤ RC < 0.48 | 50 (44.25) | 2.56 (1.44, 4.56) | 0.001 | 2.60 (1.45, 4.66) | 0.001 | 2.60 (1.45, 4.66) | 0.001 |
| Q3: 0.48≤ RC < 0.71 | 78 (67.83) | 6.81 (3.78, 12.27) | < 0.001 | 6.66 (3.65, 12.17) | < 0.001 | 6.63 (3.61, 12.20) | < 0.001 |
| Q4: ≥ 0.71 | 81 (71.05) | 7.93 (4.36, 14.42) | < 0.001 | 7.46 (4.04, 13.77) | < 0.001 | 7.41 (3.94, 13.92) | < 0.001 |
| *P* for trend | — | < 0.001 | — | < 0.001 | — | < 0.001 | — |
| **TG/HDL-C** |  |  |  |  |  |  |  |
| Per SD increase | — | 1.67 (1.40, 2.00) | < 0.001 | 1.59 (1.32, 1.92) | < 0.001 | 1.56 (1.29, 1.89) | < 0.001 |
| Q1: < 1.13 | 36 (31.86) | 1.00 (Ref) | — | 1.00 (Ref) | — | 1.00 (Ref) | — |
| Q2: 1.13 ≤ ratio < 1.71 | 56 (49.56) | 2.10 (1.22, 3.61) | 0.007 | 2.03 (1.17, 3.52) | 0.011 | 2.02 (1.17, 3.50) | 0.012 |
| Q3: 1.71 ≤ ratio < 2.69 | 63 (55.75) | 2.69 (1.57, 4.64) | < 0.001 | 2.45 (1.40, 4.29) | 0.002 | 2.38 (1.35, 4.18) | 0.003 |
| Q4: ≥ 2.69 | 80 (70.80) | 5.19 (2.94, 9.14) | < 0.001 | 4.45 (2.47, 8.00) | < 0.001 | 4.20 (2.29, 7.69) | < 0.001 |
| *P* for trend | — | < 0.001 | — | < 0.001 | — | < 0.001 | — |
| **TC/HDL-C** |  |  |  |  |  |  |  |
| Per SD increase | — | 1.69 (1.41, 2.01) | < 0.001 | 1.61 (1.34, 1.91) | < 0.001 | 1.58 (1.31, 1.92) | < 0.001 |
| Q1: < 3.64 | 39 (34.51) | 1.00 (Ref) | — | 1.00 (Ref) | — | 1.00 (Ref) | — |
| Q2: 3.64 ≤ ratio < 4.40 | 53 (46.90) | 1.68 (0.98, 2.86) | 0.059 | 1.57 (0.91, 2.71) | 0.104 | 1.55 (0.90, 2.68) | 0.118 |
| Q3: 4.40 ≤ ratio < 5.30 | 59 (52.21) | 2.07 (1.21, 3,54) | 0.008 | 1.86 (1.07, 3.24) | 0.028 | 1.81 (1.03, 3.17) | 0.039 |
| Q4: ≥ 5.30 | 84 (74.34) | 5.50 (3.10, 9.75) | < 0.001 | 4.79 (2.63, 8.71) | < 0.001 | 4.55 (2.46, 8.45) | < 0.001 |
| *P* for trend | — | < 0.001 | — | < 0.001 | — | < 0.001 | — |
| **LDL-C/HDL-C** |  |  |  |  |  |  |  |
| Per SD increase | — | 1.55 (1.30, 1.84) | < 0.001 | 1.48 (1.23, 1.77) | < 0.001 | 1.44 (1.19, 1.74) | < 0.001 |
| Q1: < 2.25 | 42 (37.17) | 1.00 (Ref) | — | 1.00 (Ref) | — | 1.00 (Ref) | — |
| Q2: 2.25 ≤ ratio < 2.91 | 54 (47.79) | 1.55 (0.91, 2.63) | 0.107 | 1.48 (0.86, 2.54) | 0.155 | 1.45 (0.84, 2.49) | 0.177 |
| Q3: 2.91 ≤ ratio < 3.58 | 59 (52.21) | 1.85 (1.09, 3.14) | 0.023 | 1.62 (0.93, 2.82) | 0.088 | 1.53 (0.87, 2.68) | 0.142 |
| Q4: ≥ 3.58 | 80 (70.80) | 4.10 (2.35, 7.15) | < 0.001 | 3.61 (2.01, 6.50) | < 0.001 | 3.36 (1.85, 6.12) | < 0.001 |
| *P* for trend | — | < 0.001 | — | < 0.001 | — | < 0.001 | — |
| **ApoB/ApoA** |  |  |  |  |  |  |  |
| Per SD increase | — | 1.59 (1.34, 1.90) | < 0.001 | 1.56 (1.30, 1.87) | < 0.001 | 1.53 (1.27, 1.84) | < 0.001 |
| Q1: < 0.69 | 43 (38.05) | 1.00 (Ref) | — | 1.00 (Ref) | — | 1.00 (Ref) | — |
| Q2: 0.69 ≤ ratio < 0.85 | 45 (39.82) | 1.08 (0.63, 1.84) | 0.785 | 0.98 (0.57, 1.71) | 0.952 | 0.98 (0.56, 1.71) | 0.948 |
| Q3: 0.85 ≤ ratio < 1.03 | 70 (61.95) | 2.65 (1.55, 4.53) | <0.001 | 2.42 (1.39, 4.20) | 0.002 | 2.34 (1.34, 4.08) | 0.003 |
| Q4: ≥ 1.03 | 77 (68.14) | 3.48 (2.01, 6.03) | < 0.001 | 3.17 (1.79, 5.63) | < 0.001 | 3.04 (1.71, 5.42) | < 0.001 |
| *P* for trend | — | < 0.001 | — | < 0.001 | — | < 0.001 | — |

Unadjusted model: no covariates applied; Model 1: adjusted for sex, age, BMI, hypertension, diabetes, smoking and alcohol intake. Model 2: Model 1+adjusted for hyperlipidemia. RC, remnant cholesterol; TG/HDL-C, the ratio of triglycerides divided by high-density lipoprotein cholesterol. The calculation methods for other lipid ratios were similar. OR, odds ratio; CI, confdence interval; SD, standard deviation; Q, quintile; Ref, refreference. Other abbreviations as in Table 1.
